# Xin-Li-Fang efficacy and safety for patients with chronic heart failure: A study protocol for a randomized, double-blind, and placebo-controlled trial

**DOI:** 10.3389/fcvm.2023.1103548

**Published:** 2023-01-27

**Authors:** Tong Liu, Sijie Yao, Wei Jiang, Taohua Lan, Wenjing Xu, Haiming Cao, Ping Yao, Chao Wang, Weihui Lu, Xiankun Chen

**Affiliations:** ^1^State Key Laboratory of Dampness Syndrome of Chinese Medicine, The Second Affiliated Hospital of Guangzhou University of Chinese Medicine, Guangzhou, China; ^2^Department of Cardiology, Guangdong Provincial Hospital of Chinese Medicine, Guangzhou, China; ^3^The Second Clinical Medical College of Guangzhou University of Chinese Medicine, Guangzhou, China; ^4^Academician Chen Keji Workstation, The Second Affiliated Hospital of Guangzhou University of Chinese Medicine, Guangzhou, China; ^5^Key Unit of Methodology in Clinical Research, Guangdong Provincial Hospital of Chinese Medicine, Guangzhou, China

**Keywords:** Traditional Chinese Medicine, protocol, chronic heart failure, randomized controlled trial, Xin-Li-Fang

## Abstract

**Introduction:**

Xin-Li-Fang (XLF), a representative Chinese patent medicine, was derived from years of clinical experience by academician Chen Keji, and is widely used to treat chronic heart failure (CHF). However, there remains a lack of high-quality evidence to support clinical decision-making. Therefore, we designed a randomized controlled trial (RCT) to evaluate the efficacy and safety of XLF for CHF.

**Methods and design:**

This multicenter, double-blinded RCT will be conducted in China. 300 eligible participants will be randomly assigned to either an XLF group or a control group at a 1:1 ratio. Participants in the XLF group will receive XLF granules plus routine care, while those in the control group will receive placebo granules plus routine care. The study period is 26 weeks, including a 2-week run-in period, a 12-week treatment period, and a 12-week follow-up. The primary outcome is the proportion of patients whose serum NT-proBNP decreased by more than 30%. The secondary outcomes include quality of life, the NYHA classification evaluation, 6-min walking test, TCM symptom evaluations, echocardiography parameters, and clinical events (including hospitalization for worsening heart failure, all-cause death, and other major cardiovascular events).

**Discussion:**

The results of the study are expected to provide evidence of high methodological and reporting quality on the efficacy and safety of XLF for CHF.

**Clinical trial registration:**

Chinese Clinical Trial Registration Center (www.chictr.org.cn). The trial was registered on 13 April 2022 (ChiCTR2200058649).

## 1. Introduction

Chronic Heart Failure (CHF) is a clinical syndrome of abnormal cardiac structure and/or function with various causes. It leads to the decline of cardiac output, systolic and/or diastolic dysfunction, metabolic derangements, and myocardial cell death (1). In China, 1.5–3.5% of the population suffers from heart failure (2). However, with the development of modern medicine, the HF survival rate is gradually rising, while the morbidity, mortality and hospitalization rates caused by HF have either remain unchanged or worsened (2).

Traditional Chinese medicine (TCM) has immense potential in improving cardiac systolic and diastolic function, patient quality of life, and other factors, and can be considered a supplementary and alternative therapeutic strategy for CHF treatment (3). Through the analysis of TCM syndrome and medication rules for CHF, it has been shown that a syndrome called Qi deficiency and Blood stasis and Water-Dampness retention (QBWD) is the most common syndrome for patients with heart failure, throughout the entire course of the disease (4, 5).

Xin-Li-Fang (XLF), as a representative Chinese patent medicine, is derived from years of clinical experience from academician Chen Keji, and is widely used to treat the CHF associated with QBWD syndrome. Details on XLF are listed in Table 1. XLF is composed of *Plantaginis Herba* (Che Qian Cao), *Curcumae Rhizoma* (E Zhu), *Ginseng Radix Et Rhizoma Rubra* (Hong Shen), *Astragali Radix* (Huang Qi), *Astragali Radix Praeparata Cum Melle* (Zhi Huang Qi) and *Corni Fructus* (Shan Zhu Yu).

**Table 1:** Components of Xin-Li-Fang.

**No**.	**Chinese name/*pinyin***	**Latin scientific name/scientific name**	**Part and form used**	**Place of origin (China)**
1	车前草/ Che Qian Cao	*Plantaginis Herba/ Plantago asiatica L*.	Dried whole grass	Anhui
2	莪术/ E Zhu	*Curcumae Rhizoma/ Curcuma kwangsiensis S. G. Lee et C. F. Liang*	Dried rhizome	Guangxi
3	红参/ Hong Shen	*Ginseng Radix Et Rhizoma Rubra/ Panax ginseng C. A. Mey*.	Dried root and rhizome	Dongbei
4	黄芪/ Huang Qi	*Astragali Radix/ Astragalus membranaceus (Fisch.) Bge.var.mongholicus (Bge.) Hsiao*	Dried root	Gansu
5	炙黄芪 Zhi Huang Qi	*Astragali Radix Praeparata Cum Melle/ Astragalus membranaceus (Fisch.) Bge.var.mongholicus (Bge.) Hsiao*	Dried root	Gansu
6	山茱萸/ Shan Zhu Yu	*Corni Fructus/ Cornus officinalis Sieb. et Zucc*.	Dried and ripe pulp	Henan

Our research team's previous studies have shown that in a rat model of CHF, XLF can improve heart function and increase the proportion of regulatory T (Treg) cells in the spleen and the lymph (this research has not been published). Meanwhile, it can also reduce the level of serum inflammatory factors and inflammatory body-related proteins, down-regulate the expression of Treg-related pathway proteins in myocardial tissue, decrease the proportion of ventricular fibrosis area, and play the role of anti-myocardial fibrosis in CHF rats (this research has also not been published).

However, there is still a lack of high-quality clinical evidence with endpoint events and adverse reactions as evaluation indexes, and the efficacy and safety of XLF remains unclear. Therefore, we designed a multicenter, double-blind, randomized placebo-controlled trial to investigate the effect and safety of XLF for CHF.

## 2. Methods

### 2.1. Design and settings

This study is a multicenter, randomized, double-blind, placebo-controlled clinical trial in which patients will be recruited from the outpatient cardiology departments at seven centers in China. The sites include Guangdong Provincial Hospital of Chinese Medicine, Xiyuan Hospital of CACMS, First Teaching Hospital of Tianjin University of Traditional Chinese Medicine, Shuguang Hospital of Shanghai University of Traditional Chinese Medicine, The Affiliated Hospital of Shandong University of Traditional Chinese Medicine, The First Affiliated Hospital of Henan University of Chinese Medicine and Xinjiang Uygur Autonomous Region Hospital of Traditional Chinese Medicine. Eligible participants with CHF will be randomly assigned to receive either XLF or a placebo for 12 weeks, and then will be followed for 12 weeks.

**Figure 1:**
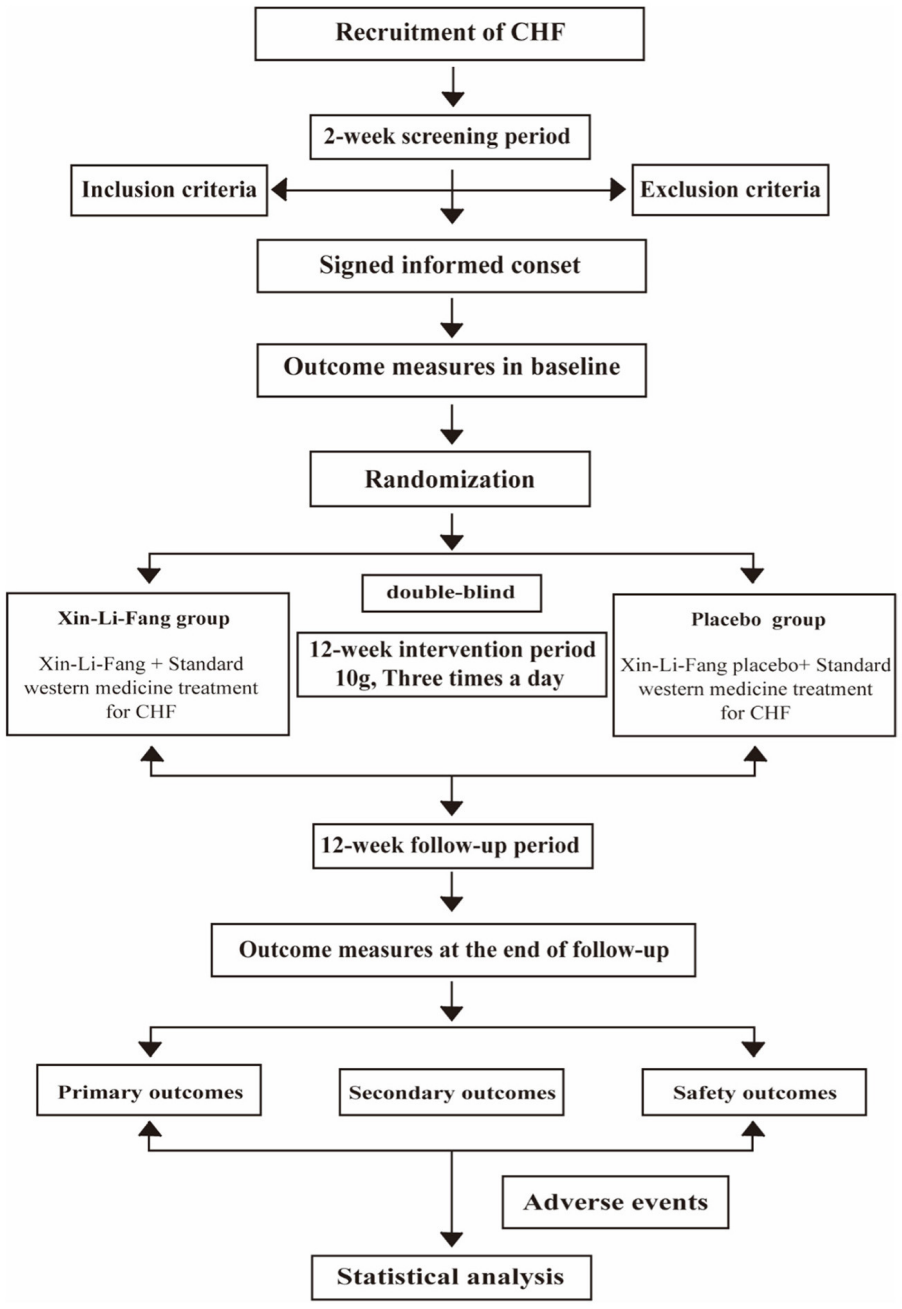
Study flowchart. CHF, chronic heart failure.

Figure 1 is a flowchart of the study design. The trial has been approved by the Ethics Committee at Guangdong Provincial Hospital of Chinese Medicine (BF2021-261-01) and registered with an identifier (ChiCTR2200058649) in the Chinese Clinical Trial Registry. Additionally, a Standard Protocol Items: Recommendations for Interventional Trials (SPIRIT) figure for the schedule of enrolment, interventions, and assessments is presented in Table 2.

**Table 2:** Study procedure table.

**Study phase time**	**Baseline**	**Intervention period**			**Follow-up**
	**−2 ~0 wks**	**4 wks**	**8 wks**	**12 wks**	**24 wks**
**Data collection at baseline**					
Inclusion/exclusion criteria	**√**				
Informed consent	**√**				
Demographic data	**√**				
Random number	**√**				
Medical history	**√**				
**Outcome measurements**					
NT-proBNP	**√**			**√**	
Echocardiography	**√**			**√**	
NYHA class	**√**	**√**	**√**	**√**	**√**
6MWT	**√**	**√**	**√**	**√**	
MLHFQ score	**√**	**√**	**√**	**√**	**√**
TCM symptom scores	**√**	**√**	**√**	**√**	**√**
Clinical-endpoint events	**√**	**√**	**√**	**√**	**√**
**Safety evaluation**					
Vital signs	**√**			**√**	
Physical examination	**√**			**√**	
Blood routine	**√**			**√**	
Urine routine	**√**			**√**	
ECG	**√**			**√**	
Blood biochemistry	**√**			**√**	
Chest radiograph	**√**			**√**	
**Other data**					
Biological sample	**√**			**√**	
Combined medications	**√**	**√**	**√**	**√**	**√**
Dispense drug	**√**	**√**	**√**		
Recovery drug		**√**	**√**	**√**	
Record AEs		**√**	**√**	**√**	**√**
Complications		**√**	**√**	**√**	
Compliance		**√**	**√**	**√**	**√**

NYHA, New York Heart Association; 6MWT, 6-min walk distance test; MLHFQ, Minnesota Living with Heart Failure Questionnaire; TCM, traditional Chinese Medicine; ECG, electrocardiogram; AE, adverse event.

“√” Represents the need for implementation at this point in time.

### 2.2. Participants

#### 2.2.1. Diagnostic criteria for CHF

(1) The CHF diagnostic criteria are based on the 2018 Chinese Guidelines for the Diagnosis and Treatment of Chronic Heart Failure published by the Chinese Medical Association Cardiovascular Disease Branch (6), 2021 ESC Guidelines for the diagnosis and treatment of acute and chronic heart failure (7), and the 2022 AHA/ACC/HFSA Guidelines for the Management of Heart Failure (8).

(2) Patients must have a history of CHF of at least 3 months, or clinical symptoms of HF for at least 3 months.

(3) Patients must have received standard medical treatment for at least 2 weeks and no modification of dosage or intravenous administration.

#### 2.2.2. Diagnostic criteria for “Qi deficiency and Blood stasis and Water-Dampness retention (QBWD)” syndrome

QBWD syndrome diagnosis refers to expert consensus on CHF diagnosis and treatment in Chinese medicine, and textbooks on internal medicine for integrated traditional Chinese and Western medicine (9, 10). Syndrome differentiation will be independently determined by two qualified TCM cardiologists according to the diagnostic criteria for TCM differentiation (Table 3).

**Table 3:** Diagnostic criteria for traditional Chinese medicine syndromes.

**Qi deficiency and blood stasis and water-dampness retention (QBWD)**	
Main symptoms	1. Shortness of breath
	2. Weakness
	3. Palpitations
Qi deficiency syndrome	1. Palpitations and shortness of breath
	2. Fatigue
	3. Spontaneous sweating
	4. Pale complexion
	5. Pale tongue
	6. Corpulent tender tongue with indentations in the margin of the tongue
	7. Weak pulse
	The included patients need to have two or more of the above symptoms
Blood stasis syndrome	1. Dark purple lips, face or hands
	2. Purple, dark or bruised tongue
	3. Enlarged sublingual venation
	4. Uneven pulse
	The included patients need to have two or more of the above symptoms
Water-dampness retention syndrome	1. Edema
	2. Hydrothorax/Ascites
	3. Poor urination
	The included patients need to have two or more of the above symptoms

#### 2.2.3. Inclusion criteria

Patients who meet all of the following criteria will be enrolled:

1. At least 18 years of age
2. Meet the CHF diagnosis criteria
3. QBWD syndrome diagnosis
4. New York Heart Association (NYHA) classification stages II-IV
5. Serum NT-proBNP≥450 pg/ml (11)
6. Signed informed consent.

#### 2.2.4. Exclusion criteria

Patients with one of the following conditions will be excluded:

(1) CHF caused by any of the following conditions: valvular disease, congenital heart disease, pericardial disease, cardiac arrhythmia, or other non-cardiogenic factors, including dysfunction in the kidneys, liver or lungs.

(2) Severe liver, kidney, hematological disease or abnormal renal or hepatic function. Patients with tumors, severe neural, endocrine or psychological disease; judgment from an investigator predicting that the survival period will not exceed 1 year.

(3) Comorbid with left ventricular outflow tract obstruction, myocarditis, hypertrophic cardiomyopathy, restrictive cardiomyopathy, aortic aneurysm, aortic dissection, congenital heart disease, or significant hemodynamic changes in patients with silent valvular heart disease; patients with cardiogenic shock, uncontrollable malignant arrhythmia, sinus or atrioventricular block without pacemaker treatment, progressive unstable angina pectoris or acute myocardial infarction.

(4) Patients undergoing cardiac resynchronization therapy, or who will undergo coronary revascularization or cardiac resynchronization therapy within 12 weeks.

(5) Patients with hypertension without control, SBP ≥ 180 mmHg and/or DBP ≥ 110 mmHg; SBP < 90 mmHg and/or DBP < 60 mmHg.

(6) Patients with allergies, or those who are known to be allergic to this medicine and its ingredients.

(7) Patients who have been involved in any clinical trial within the past month.

(8) Pregnant or lactating women.

(9) Investigator judges that patient could not complete the study or could not comply with the requirements of the study.

### 2.3. Recruitment process

Participants will be recruited *via* on-site screening of outpatients and inpatients, regular screening of potential participants using electronic medical records, and referrals from physicians and other providers. Patients who meet the inclusion criteria and sign informed consent forms will enter the screening period; otherwise, they will be excluded before randomization. Patients who agree to participate will be examined and diagnosed jointly by the main investigator and their attending physician according to the inclusion and exclusion criteria to determine their eligibility for the trial. Patients who pass the screening and sign the informed consent will be enrolled in the online allocation system and subsequently participate in the experiment's intervention and follow-up periods. In addition, the demographic characteristics of any ineligible patients and the reasons for not participating will be recorded. All personal patient information will be kept strictly confidential.

### 2.4. Randomization and allocation concealment

Through the central randomization system, the patients will be randomly divided into a Xin-Li-Fang group and a placebo group at a 1:1 ratio. Stratified by the central stratification method, we will use SAS 9.2 statistical software to generate random numbers and the random assignment results. The investigators will obtain the randomized number and drug number by a sequential number in the central randomization system. All center numbers, sequence numbers, randomization numbers, and drug numbers will be managed by the statistical unit.

### 2.5. Blinding

This study is a double-blind trial, in which neither participants nor study personnel will know the group allocation or be able to identify the treatment. Both the XLF and placebo granules will be manufactured by the Jiangyin Tianjiang Pharmaceutical Co., Ltd. (Jiangyin, China), and the placebo will be identical to the SGR in color, size, shape, and taste. All of the above drugs comply with the requirements of the National Drug Manufacturing Code of China. Blinding codes will be assigned after the randomization operation. This process will be operated by a specially assigned person, and the sequence number of the subject, corresponding random numbers and grouping results (i.e., the subjects are assigned to either a Group A or a Group B) will be the primary blinding base. Then, the two groups will be blinded to the medication, which will be the secondary blinding base (i.e., which of Group A or Group B used the TCM and which used the placebo), and each subject's drug number will be randomly prepared in order. All operations will be recorded and properly stored. The statistician will uncover the blinding when necessary, in case any serious adverse events (SAEs) occur during the course of the study, or emergency care is needed in an emergency situation. Once unblinded, the participant will be withdrawn from the study and the investigator will report the reason to the examiner within 24 h.

### 2.6. Interventions

#### 2.6.1. Routine care

Referring to the 2018 Chinese Guidelines for the Diagnosis and Treatment of CHF (6), the subjects will be given conventional basic treatment to reduce cardiac load and reverse ventricular remodeling, including the use of ACEIs, ARBs, ARNIs, diuretics, β-blockers and other standardized drug therapies. After entering the treatment period, the type and dose of the drugs used for each patient will continue the standard treatment plan before enrollment. If the patient needs to adjust the drug dosage or type due to a change in condition during the treatment period, the drug name, dosage, frequency and time of use, the composite endpoint events and adverse events will be recorded in the CRF table.

#### 2.6.2. Experimental group

Patients in the experimental group will receive routine care plus XLF granules (2 bags at 10 mg each, orally, three times a day, with warm water) for 12 weeks, and the total observation follow-up period will be 24 weeks.

#### 2.6.3. Control group

Patients in the control group will receive routine care plus placebo granules (2 bags at 10 mg each, orally, three times a day, with warm water) for 12 weeks and the total observation follow-up period will be 24 weeks.

#### 2.6.4. Concomitant medication during the trial

During the trial, we do not suggest using other similar TCM with the goal of invigorating qi or promoting blood circulation and diuresis, or any Chinese patent medicines for HF that are stated in the instructions. Concomitant therapy for comorbidities will be allowed during the trial.

#### 2.6.5. Monitoring compliance

The subjects' compliance will be evaluated by recording the distribution and recovery of the medication. Patients with compliance rates equal to or >80% will be considered as having high compliance. Any reasons for not taking the medication will be recorded as well.

### 2.7. Outcome measurements

The time points of the study data collection for outcome measurements are shown in Table 2.

#### 2.7.1. Primary outcomes

The primary outcome is the proportion change in patients with a decrease in serum NT-proBNP of more than 30% after 12 weeks of treatment.

#### 2.7.2. Secondary outcomes

The secondary outcomes will include: (1) Minnesota Living with Heart Failure questionnaire (12); (2) NYHA classification evaluation (13); (3) 6-min walking test (14); (4) TCM symptoms evaluation; (5) the Evaluation Scale of Dampness Syndrome of Traditional Chinese Medicine (15); (6) echocardiography parameters, and (7) clinical events (e.g., hospitalization for worsening heart failure, all-cause death, other major cardiovascular events).

### 2.8. Safety assessments

Information on adverse events (AEs), including the occurrence time, severity, duration, adopted measures, and prognosis, will be recorded truthfully and in detail. Additionally, the investigator will assess every AE recorded to analyze the causality between the AE and the studied drugs. Serious adverse events (SAEs) are defined as events which can cause hospitalization, loss of ability to work, disability, congenital deformity, or death, according to the International Council for Harmonization of Technical Requirements for Pharmaceuticals for Human Use (ICH) guidelines (16). Besides the abovementioned measures, in the incidence of any SAE, the principal investigator will be notified. Additionally, a report will be submitted to the ethics committee and the data and safety monitoring committee (DSMC) within 24 h.

### 2.9. Sample size calculation

Sample size calculation is based on the level of NT-proBNP, the main efficacy indicator. The literature showed that after 12 weeks of conventional basic treatment combined with placebo, NT-proBNP levels decreased by more than 30% in 31.98% of the patients, and after 12 weeks of conventional basic treatment combined with TCM, NT-proBNP levels decreased by more than 30 in 47.95% of the patients (17). Therefore, we estimate that after 12 weeks of conventional basic treatment combined with XLF, the NT-proBNP levels will decrease by more than 30 in 50% of the patients. Thus, assuming α = 0.05 and β = 0.20, the calculated sample size required for each group is 114 cases, after substituting the above values in PASS 11. Considering that 20% will miss visits, the sample size is adjusted to 150 cases in each group.

### 2.10. Statistical analysis

Statistical analysis will be conducted with SAS 9.2 (SAS Institute Inc., USA) in accordance with a pre-established statistical analysis plan. Data analysis for efficacy will be performed following the intention-to-treat (ITT) principle. The full analysis set (FAS) comprises all randomly assigned patients, but rejects those who are wrongly enrolled, or do not receive any assigned treatment or follow-up. The per-protocol set (PPS) includes patients who complete the planned treatment program. The safety set (SS) includes patients who received at least one treatment after randomization and were evaluated for safety. Efficacy analysis will be performed on the FAS and PPS. All baseline demographics will be analyzed on the FAS, and safety evaluation will be performed on the SAS.

The two groups' baseline characteristics will be summarized by descriptive statistics. For the primary and secondary outcomes, a *t*-test or Mann–Whitney *U* test will be employed for continuous variables with normal or unknown distributions; Chi-squared test or Fisher's exact test will be used for categorical variables. Any change across time among the primary or secondary outcomes will be analyzed by the generalized estimated equation model or a mixed-effects model, as appropriate. Sub-group analysis will also be conducted according to the pre-established statistical analysis plan.

### 2.11. Data management

Data management will ensure the authenticity, integrity and accuracy of clinical trial data, and the data management process will comply with regulatory requirements such as Good Clinical Practice (GCP) (18) and the Technical Guidelines for Clinical Trial Data Management (19), and ensure the traceability of clinical trial data.

### 2.12. Quality control

Researchers will perform their duties, follow the clinical research plan, adopt standard operating procedures, and verify all relevant observations and findings to ensure the implementation of the quality control and quality assurance system of the clinical research. In the clinical study, subject allocation must adhere to the random allocation scheme determined by the study design, and each subject's processing grouping code will be saved by the statistical unit and the researcher as the blind background.

The investigator must provide necessary training for all personnel participating in the clinical study, explain any relevant data, operation specifications or responsibilities, and ensure that the data are truthfully, accurately, completely, timely and legally recorded in the medical records and CRFs. CRFs must be kept by special personnel.

The supervisor will follow the standard operating procedures, promote the implementation of the research plan, and confirm that all data records and reports are correct and complete, and that all CRF are correctly filled out and consistent with the original data. The supervisor will systematically check the activities and documents related to the clinical study to evaluate whether the study has been conducted in accordance with the protocol, standard operating procedures and relevant laws and regulations.

All clinical laboratory indexes must be accurately recorded, and a copy of the original report needs to be pasted on the case report form. In order to check the data quality and implementation process, the medical statisticians will input the research data into the report comprehensively and correctly, and all steps involved in data management will be recorded. The clinical research will accord with the approved protocol, and any deviation from the protocol will be recorded. Any modification of the research scheme needs to be described and reported to the Ethics Committee for approval before implementation.

### 2.13. Ethical considerations

This trial will be conducted in accordance with the principles of good clinical practice and the Declaration of Helsinki. Before signing informed consent, all participants will be informed of the purpose, interventions, possible benefits and harm of this study. Completing the informed consent form and qualifying for the trial will not affect patients' subsequent treatment, regardless of when or why they withdraw.

Patients will have the right to withdraw from the study at any time for any reason, but should try to avoid unnecessary withdrawal. They should take measures to complete follow-up as far as possible, so that its efficacy and safety can be evaluated. However, if a patient decides to withdraw, the researcher must contact the patient or his responsible relatives by telephone or personal interview, and confirm the reason for withdrawal if possible. The researcher will retrieve the remaining drugs when the patient withdraws, and complete the final evaluation and the case report as much as possible, explain the reason for withdrawal, and follow up on the occurrence of the endpoint event for the withdrawing patient. If the reason for patient withdrawal is an adverse event, this must be recorded in the complete CRF.

## 3. Discussion

XLF, as an empirical prescription of academician Chen Keji, has long been used as a clinical treatment for CHF. This trial is the first randomized, double-blind, placebo-parallel controlled experiment conducted in China to investigate the safety and clinical efficacy of XLF, so as to provide high-quality clinical evidence for optimizing the CHF treatment scheme.

Most TCM is used to achieve multiple therapeutic effects in the form of compounded Chinese medicine (20, 21). However, standardizing clinical randomized controlled trials on compound Chinese medicine has presented several challenges (22). For example, it is difficult to conduct clinical research under the guidance of “syndrome differentiation and treatment” in TCM theory (23). To mitigate influence from patients with other symptoms, in this study, based on the current analysis of TCM syndrome and medication rules of CHF, we chose the most common syndrome “Qi deficiency, Blood stasis and Water and Dampness retention (QBWD).” Additionally, diagnosis of QBWD and CHF have been clarified by the latest guidelines and textbooks. To ensure enrollment accuracy, diagnoses and examinations will be done collaboratively by the principal investigators and their attending physicians. Additionally, the measures of double blinding and random grouping will be implemented to prevent bias. Patients' grouping results and medication numbers will be blinded by specially assigned personnel. Meanwhile, an XLF placebo will be produced, the appearance, taste and weight of which will be the same as the test drug, to ensure that neither researchers nor patients are aware of the drug properties. The above measures can guarantee the authenticity and reliability of the data collection and the evaluation of the results.

Heart failure can be divided into three types: HFrEF, HFpEF, and HFmrEF. Each type has different treatment plans. Xin-Li-Fang is a compound traditional Chinese medicine preparation. There are many effective ingredients in the preparation. Heart failure treatments may also have multiple targets and a complex mechanism. At present, according to our clinical observations, Xin-Li-Fang has three types of therapeutic effects. Therefore, we did not distinguish the types of heart failure in the overall design of the trial, but included them non-selectively to avoid overlooking more possibilities of Xin-Li-Fang in the field of heart failure treatment. However, we plan to conduct a subgroup analysis on patients with different types of heart failure to further explore whether Xin-Li-Fang has superior efficacy on patients with a certain type of heart failure.

In our clinical practice, it has been noted that Xin-Li-Fang has an effect on the stable and aggravating stages of CHF. The heart failure patients with NYHA Classification Stage I are not the object of our study because it is difficult to make significant differences in the efficacy indicators before and after treatment. At the same time, studies have shown that most patients have similar self-perceptions of their limitations, objective physical capabilities, and levels of natriuretic peptides between NYHA Classes I and II (24). This may be due to the substantial overlap in the basic characteristics of most mild heart failure patients which may mitigate the clinical relevance of these statistical group differences (25). Therefore, we hope to scientifically confirm that patients with heart failure in NYHA Classes II-IV are likely to benefit from Xin-Li-Fang treatment. To avoid the high likelihood of selection bias, patients with acute heart failure due to acute decompensation or other causes, acute exacerbation of chronic heart failure or unstable NYHA Class IV chronic heart failure were excluded in our exclusion criteria design. We did this to ensure that the baseline level of the included patients was as consistent as possible.

Heart failure is the end-stage manifestation of many heart (and non-heart) diseases. There are various primary diseases that may cause heart failure. In this study, we first established the inclusion and exclusion criteria to screen patients with cardiogenic heart failure. However, patients with cardiogenic heart failure also have many primary diseases which lead to heart failure, such as ischemic heart disease, hypertensive heart disease and arrhythmia. Our previous clinical observation of Xin-Li-Fang showed that Xin-Li-Fang is a compound traditional Chinese medicine preparation with a variety of effective components, and the treatment for heart failure may also be a multi-target and compound mechanism. It has been observed that Xin-Li-Fang may have an effect on heart failure caused by a variety of primary diseases. Therefore, the overall design of our trial did not stratify the primary cause of heart failure, but only focused on cardiogenic heart failure. We did this so that we would not overlook more possibilities of Xin-Li-Fang in the field of heart failure treatment. In subsequent trial analysis, we plan to conduct a subgroup analysis of different causes to further explore whether Xin-Li-Fang has a better effect on heart failure caused by certain primary diseases.

In our study, using a proportion of patients whose serum NT-proBNP had decreased by more than 30% was an innovative design. The serum NT-proBNP concentration in patients with heart failure is high, and this is influenced by renal clearance rate. Additionally, the basal NT-proBNP levels in heart failure patients with different ages and different NYHA classifications varies greatly. Thus, the individual differences in the serum NT-proBNP concentration in included patients are considerable. Based on the above characteristics of NT-proBNP, the baseline NT-proBNP of the patients included in the trial may range from several hundred to tens of thousands. If the decrease in NT-proBNP is directly used as an efficacy indicator for comparison between groups, there may be wide disparities between groups, baseline imbalance, and no comparability. However, patients with a higher NT-proBNP baseline may have a more significant reduction after Xin-Li-Fang intervention, which may lead to a large 95% confidence interval in the outcome indicators, thus preventing the scientific evaluation of Xin-Li-Fang's efficacy and safety.

In this study, the proportion of patients whose serum NT-proBNP decreased by more than 30% serves as the primary outcome. NT-proBNP is a cardiovascular neurohormone secreted by ventricular myocytes, which can be used for the diagnosis and differential diagnosis of HF, risk stratification, and prognosis evaluation (26). As the best biomarker in the laboratory, NT-proBNP is easy to measure, accurate and repeatable, and often used to evaluate the clinical efficacy of drugs (27). In addition, NT-proBNP is a sensitive marker for the diagnosis of heart failure, and its level is more than 450 ng/ml; the accuracy of determining patients with CHF can be as high as 96% (11). Guidelines recommend NT-proBNP levels as the most valuable and reliable biomarker for diagnosing heart failure (28). They are used in many diagnoses, risk classifications, and prognostic evaluation of CHF. Studies have shown that CHF-related death risk is positively correlated with NT-proBNP level (29). Additionally, a previous study has suggested that the prognosis of patients with NT-proBNP level decreasing by at least 30% from baseline after treatment is significantly better than that of patients with no significant change or increased NT-proBNP level (30). Therefore, we selected the proportion of patients with NT-proBNP that decreased by more than 30% in serum as the primary outcome for this study.

6MWT can reflect the cardiac function of patients from exercise tolerance. The method is simple, safe, easy to perform, and reproducible, and widely used in systematic reviews of the treatment of CHF with TCM (14, 31). Echocardiography plays a key role in the diagnosis and classification of CHF. Among the various echocardiography indicators, LVEF is the first choice for evaluating left ventricular systolic function (32). Moreover, left ventricular end systolic diameter (LVSDD), left ventricular end diastolic diameter (LVEDD) and inlet ventricular septal thickness (IVST) are the most common indicators for CHF systematic evaluation (32). Additionally, in the systematic evaluation of TCM treatment for CHF, TCM symptoms evaluation, the Minnesota Living with Heart Failure Questionnaire, and improvement of NYHA classification are often used as the evaluation criteria of clinical efficacy (32). Monitoring adverse events and adverse reactions are of great significance for evaluating drug efficacy and guiding clinical medication (33).

Regardless, several potential limitations of this study should be considered. Firstly, although we will recruit patients with “qi deficiency, blood stasis and water and dampness retention” syndrome, this Chinese medicine syndrome can dynamically change after the intervention. Thus, patients may take the XLF while the TCM syndrome is changing, and this will not be considered in our study. Secondly, sample size calculation in our study will be based on existing literature, and it should be recalculated after our pilot study. Thirdly, we will investigate the effect of XLF combined with conventional basic therapy in CHF patients, however the therapeutic effect of XLF alone will not be addressed.

In conclusion, this trial will provide high-quality evidence for XLF for CHF as a conjunctive therapy. Whether the results are neutral, negative, or positive, this trial will have clinical implications for patients with CHF.

## Ethics statement

The studies involving human participants were reviewed and approved by the Ethics Committee of Guangdong Provincial of Chinese Medicine approved this trial (BF2021-261-01). The patients/participants provided their written informed consent to participate in this study.

## Author contributions

TLi and WL designed the study. TLi and XC conceived the study and wrote the protocol. TLi and SY contributed equally to the study and wrote the manuscript. HC, PY, and WX are responsible for the quality control of the test drug. TLa and WJ participated in the modification of the study protocol. All authors read and approved the final version of the manuscript.
